# Isolation of *Neisseria meningitidis* Serogroup B from Urine in a 74-Day-Old Infant: A Case Report

**DOI:** 10.3390/microorganisms14091977

**Published:** 2026-09-07

**Authors:** Argyro Siapika, Fevronia Kolonitsiou, Venetia Bellou, Despoina Gkentzi, Nikolaos Giormezis

**Affiliations:** 1 Department of Microbiology, School of Medicine, University of Patras, 26504 Patras, Greece; 2 Department of Pediatrics, School of Medicine, University of Patras, 26504 Patras, Greece

**Keywords:** *Neisseria meningitidis*, serogroup B, invasive meningococcal disease, infant, urine culture, bacteremia, cerebrospinal fluid pleocytosis

## Abstract

*Neisseria meningitidis* is a major cause of invasive bacterial disease in infancy, usually presenting as meningitis and/or septicemia. Initial isolation of this organism from urine is exceptionally rare, particularly in young infants. We report a 74-day-old previously healthy infant who presented with fever without an apparent source. Urine obtained by sterile suprapubic aspiration unexpectedly grew *N. meningitidis* serogroup B. Blood culture subsequently confirmed invasive meningococcal disease, whereas cerebrospinal fluid (CSF) culture and multiplex PCR were negative despite pathologic CSF findings. The urinary isolate was therefore interpreted as probable secondary hematogenous seeding rather than primary urinary tract infection. The patient received ceftriaxone, with temporary escalation to meropenem because of persistent fever; neuroimaging excluded intracranial complications. Complement screening (50% hemolytic complement, CH50) was normal, and the infant recovered fully. This case illustrates an unusual microbiological presentation of probable invasive meningococcal disease, and highlights the importance of promptly interpreting unexpected culture results in febrile young infants.

## 1. Introduction

*Neisseria meningitidis* is a Gram-negative, oxidase-positive diplococcus that commonly colonizes the human nasopharynx and may cause invasive meningococcal disease (IMD), most often manifesting as meningitis and/or septicemia. Although unusual presentations such as arthritis, osteomyelitis, conjunctivitis, and chronic meningococcemia have been described, urinary tract involvement is exceptionally uncommon and has been reported mainly in adults with urethritis [1,2]. In infants younger than 3 months, fever without source (FWS) remains a common and diagnostically challenging presentation because serious bacterial infection must be excluded promptly [3].

*N. meningitidis* is classified into serogroups according to its capsular polysaccharides, with serogroups A, B, C, W, X, and Y accounting for most invasive disease worldwide [4]. Its virulence is related to the polysaccharide capsule, outer membrane proteins, and lipooligosaccharide, which contribute to immune evasion, bloodstream survival, and host tissue injury [5]. Humans are the only reservoir, and transmission occurs through respiratory droplets. Despite major progress in vaccination, IMD continues to cause substantial morbidity and mortality globally [6]. In Europe, serogroup B remains the leading cause of IMD, particularly in infants and young children, who have the highest disease burden [7,8,9,10]. In this age group, clinical presentation is often non-specific, which may delay diagnosis and treatment [8].

FWS is frequent in young infants. Although viral infections account for the majority of cases, occult bacteremia and meningitis remain important considerations, especially during the first 90 days of life [11,12]. Accordingly, unexpected microbiological findings from normally sterile or carefully collected specimens should be interpreted cautiously and in the context of overall clinical status.

We describe a 74-day-old infant with IMD due to *N. meningitidis* serogroup B in whom the organism was first isolated from urine obtained by sterile suprapubic aspiration. To our knowledge, this represents a highly unusual presentation and raises important diagnostic considerations regarding secondary urinary tract seeding during meningococcal bacteremia.

## 2. Case Description

A 74-day-old previously healthy term female infant presented to the Pediatric Emergency Department of the University Hospital of Patras, Greece with a 24-h history of fever (38.6 °C) and irritability. Parents reported decreased feeding over the previous day and nasal congestion for three days. There were no lower-respiratory symptoms and no known exposure to infectious diseases. The infant was born via cesarean section and had no significant personal or family medical history, except for a hospitalization one month earlier due to bronchiolitis. She had not yet received any vaccinations. There was no history of gastrointestinal or urinary symptoms.

On examination, the infant appeared well. Vital signs were as follows: heart rate 144 beats per minute, blood pressure 102/57 mmHg, temperature 38.6 °C, and oxygen saturation 99% on room air. Physical examination was unremarkable. A chest X-ray revealed mild thickening of the bronchial walls and peribronchial markings in the hilar regions and the left lower lobe, consistent with recent bronchiolitis. Laboratory evaluation showed a white blood cell count of 10.68 K/μL (59.8% neutrophils), hematocrit 32.2%, and a C-reactive protein (CRP) level of 7.31 mg/dL. Blood and urine cultures were obtained, the urine being collected via suprapubic aspiration. Due to the small sample volume, a urine analysis was not performed. The infant was empirically started on intravenous ceftriaxone at 100 mg/kg/day for presumed occult bacteremia, and was admitted for observation.

The infant’s clinical course is described in Table 1. On the third day of hospitalization, the white blood cell count had increased to 12.65 K/μL, and CRP had risen to 20.53 mg/dL. The urine culture showed growth of 48,000 colony-forming units (CFUs)/mL of Gram-negative cocci which were catalase and oxidase positive. Matrix-assisted laser desorption/ionization time-of-flight mass spectrometry (MALDI-TOF MS, Bruker Daltonics, Bremen, Germany) identified the organism as *Neisseria meningitidis*. Serogrouping was performed by slide agglutination using group-specific antisera, and the isolate was categorized as serogroup B.

Given the unexpected urine isolate, cerebrospinal fluid (CSF) and repeat blood cultures were obtained. CSF appeared bloody and contained 30,000 red blood cells/mm^3^ (indicating a potentially traumatic lumbar puncture) and 920 white blood cells/mm^3^ (76% polymorphonuclear cells). CSF glucose was 49 mg/dL (serum glucose, 97 mg/dL), and CSF protein was 274.6 mg/dL. Gram stain was negative, and no pathogen was detected with the FilmArray^®^ Meningitis/Encephalitis Panel (bioMérieux, Marcy-l’Étoile, France), which includes *N. meningitidis* among the bacterial targets. CSF culture remained sterile after 72 h of incubation.

Later the same day, the blood culture taken upon admission flagged positive. Gram stain again showed Gram-negative cocci, and subculture on blood agar confirmed the presence of *N. meningitidis*, identified by both MALDI-TOF MS and a VITEK 2 Compact system (bioMérieux, Marcy-l’Étoile, France), and serogrouped as type B. Antimicrobial susceptibility testing was performed for both the urine and blood isolates using E-test (bioMérieux, Marcy-l’Étoile, France), and MICs were interpreted according to the European Committee on Antimicrobial Susceptibility Testing (EUCAST) clinical breakpoints, version 15.0 (2025) [13]. The MIC values obtained for the urine and blood isolates were identical: 0.094 mg/L for benzylpenicillin, 0.023 mg/L for ampicillin, 0.003 mg/L for cefotaxime, <0.016 mg/L for ceftriaxone, 0.008 mg/L for meropenem, and 0.002 mg/L for ciprofloxacin. No separate formal quality-control strain testing was performed.

Despite three days of ceftriaxone, the infant remained febrile. At that stage, the persistent fever raised concern about a possible ongoing or additional infectious process, such as an occult bacterial focus, a concomitant infection, or an alternative source of fever that had not yet been identified. Meropenem was therefore used empirically while further clinical assessment was undertaken. Meropenem was added at 120 mg/kg/day, and a brain Magnetic Resonance Imaging (MRI) was performed to exclude an abscess. The imaging was normal. The final febrile episode occurred after three days; hence, meropenem was stopped. On hospital day 5, a repeat lumbar puncture was performed. CSF analysis showed 410 white blood cells/mm^3^, 3560 red blood cells/mm^3^, glucose 63 mg/dL, and protein 246.5 mg/dL. Gram stain and FilmArray were negative, and the culture remained sterile after 72 h. Molecular testing for other viral pathogens in the CSF was also negative.

An abdominal ultrasound, including the kidneys, ureters, and bladder, revealed no abnormalities. The infant remained afebrile and in good clinical condition. Blood cultures taken on hospital days 3 and 5 were negative. On day 11 of hospitalization, she was discharged after completing 10 days of intravenous ceftriaxone. At discharge, CRP had normalized (0.18 mg/dL), and the infant was well. To investigate possible immunodeficiency, a CH50 complement test was performed, yielding a normal result (70.2 U/mL). The normal CH50 did not suggest a major classical/terminal-pathway defect but did not exclude isolated alternative-pathway or other complement abnormalities. The final diagnosis was bacteremia with CSF pleocytosis compatible with meningitis due to *Neisseria meningitidis* serogroup B. Follow-up at 3 months after discharge demonstrated normal growth and development.

## 3. Discussion

We describe invasive meningococcal disease due to *N. meningitidis* serogroup B in a 74-day-old infant, in whom the organism was first recovered from urine obtained by suprapubic aspiration. Although *N. meningitidis* is classically associated with meningitis and septicemia, its recovery from the urinary tract is distinctly uncommon, particularly in early infancy, and requires careful clinical interpretation.

In our patient, the positive blood culture for *N. meningitidis*, the inflammatory CSF findings, and the clinical response to anti-meningococcal therapy support IMD with bacteremia and probable central nervous system involvement. Because both CSF culture and multiplex PCR were negative, the case is described more cautiously as invasive meningococcal disease with CSF pleocytosis compatible with meningitis, rather than as culture-confirmed meningococcal meningitis. This distinction matters in a case report, where the diagnostic terminology should reflect the available microbiological evidence.

The infant presented with fever without an apparent source, a scenario that warrants prompt and systematic evaluation in infants younger than 90 days because serious bacterial infection may initially appear clinically subtle [11,12]. In this setting, the isolation of *N. meningitidis* from urine collected by sterile suprapubic aspiration was particularly informative. Because this collection method minimizes contamination, and because the patient had no urinary symptoms or ultrasonographic evidence of urinary tract disease, the urine culture result was considered most compatible with, but did not prove, secondary hematogenous seeding during meningococcal bacteremia rather than a primary urinary tract infection.

A possible explanation is secondary hematogenous dissemination during meningococcal bacteremia, with transient seeding of the renal microvasculature and subsequent urinary excretion of the organism. This can result in transient bacteriuria without a primary urinary tract infection. In our case, the concomitant bacteremia, absence of urinary symptoms, and normal renal ultrasonography support this interpretation, although the precise mechanism cannot be confirmed.

Urinary isolation of *N. meningitidis* in infancy appears to be exceptionally uncommon. Published reports of genitourinary meningococcal infection have predominantly involved older patients with urethritis, whereas neonatal and infant cases of meningococcal disease have more typically presented with bacteremia and/or meningitis [14,15,16,17]. These previously described presentations differ substantially from the present case, in which *N. meningitidis* was recovered from urine obtained by suprapubic aspiration in a 74-day-old infant without urinary symptoms or evidence of urinary tract pathology. In this context, the urinary isolate is more appropriately interpreted as an unusual manifestation accompanying invasive meningococcal disease rather than as a primary genitourinary infection. The systematic review by Poletto et al. addressed concomitant bacterial meningitis in febrile young infants with suspected or confirmed urinary tract infection but did not specifically establish *N. meningitidis* as a urinary pathogen in this population [18].

Published data about infants also support the unusual nature of this presentation. By contrast, meningococcal infection in neonates and young infants usually presents as bacteremia, meningitis, or both, sometimes in association with maternal genitourinary colonization or close household exposure [17]. In a systematic review of febrile infants ≤ 3 months of age with presumed urinary tract infection who underwent lumbar puncture, the organisms recovered from urine in cases with concomitant meningitis included *Escherichia coli*, *Enterobacter* spp., *Staphylococcus aureus*, and *Klebsiella oxytoca*, but not *N. meningitidis* [18]. Against this background, our case suggests that meningococcal growth in urine, when obtained from a sterile specimen and interpreted in the appropriate clinical context, may reflect disseminated infection.

From a microbiological perspective, identification of the urine isolate by MALDI-TOF MS, followed by serogrouping with specific antisera and concordant identification of the blood isolate, strengthens the validity of the diagnosis. This is particularly relevant because differentiation from commensal *Neisseria* species can be challenging in routine practice. The combination of Gram-negative diplococcal morphology, oxidase positivity, MALDI-TOF MS identification, and confirmation from blood provided strong evidence that the organisms recovered from urine and blood were *N. meningitidis* serogroup B.

Ceftriaxone was appropriately initiated empirically in this young infant with suspected invasive bacterial infection, and was subsequently continued as definitive therapy after N. meningitidis was identified. In the present case, ceftriaxone was started promptly, consistent with recommended management of possible occult bacteremia and bacterial meningitis in young infants [19]. Meropenem was added temporarily because of persistent fever despite three days of ceftriaxone, raising concern for an ongoing or complicated infectious process, including an occult intracranial focus or other complication not yet identified. Brain MRI was performed to exclude a focal intracranial complication; both the imaging findings and subsequent clinical course were reassuring. The decision to add meropenem was, consequently, a case-specific response to the persistent fever, rather than a standard component of meningococcal disease management. Globally, antimicrobial resistance in *N. meningitidis* remains relatively uncommon, although reduced susceptibility to penicillin and increasing fluoroquinolone resistance have emerged as important concerns [20]. In contrast, third-generation cephalosporins, including ceftriaxone and cefotaxime, continue to demonstrate excellent activity against meningococci, with confirmed resistance remaining exceptionally rare.

Assessment of complement function was also appropriate because inherited deficiencies of the terminal complement pathway are associated with increased susceptibility to invasive meningococcal infection, particularly recurrent disease [21,22]. The normal CH50 result argued against a major classical or terminal complement pathway defect; however, because AH50 and additional immunological investigations were not performed, an underlying complement or other immunological abnormality could not be completely excluded.

This case also underscores the importance of keeping a broad differential diagnosis when evaluating febrile young infants, even when the initial clinical examination appears reassuring. In the absence of typical manifestations of meningococcal disease, such as petechial rash, hemodynamic instability, or significant neurological findings, invasive infection may be difficult to recognize at presentation. In such cases, microbiological investigation remains crucial, as early invasive disease may precede the development of more characteristic clinical signs. In the present case, the unexpected isolation of *N. meningitidis* served as an important diagnostic clue, prompting further evaluation and ultimately leading to the recognition of bacteremia and probable central nervous system involvement.

This case has some limitations. As a single case report, its findings cannot be generalized to all young infants with invasive meningococcal disease. In addition, although *N. meningitidis* was isolated from a normally sterile site, meningitis could not be confirmed by cerebrospinal fluid culture, and central nervous system involvement was therefore considered probable rather than definitive. Urinalysis was not performed, no repeat urine culture was obtained, and no molecular analysis of the isolates was performed. Also, AH50 or additional immunological testing was not performed. The retrospective nature of the case and the limited availability of serial clinical and microbiological data also make it difficult to fully assess the evolution of the infection and the response to treatment.

## 4. Conclusions

This case adds to the recognized clinical spectrum of invasive meningococcal disease by showing that *N. meningitidis* may rarely be recovered from urine in a young infant without evidence of primary urinary tract pathology. It underscores three practical messages: first, febrile infants younger than 3 months require careful evaluation even when they appear initially well; second, unexpected microbiological findings from sterile or carefully collected specimens should be interpreted within the full clinical context; and third, prompt initiation of appropriate antimicrobial therapy remains essential when IMD is suspected. Awareness of such atypical presentations may help facilitate earlier recognition and appropriate management of meningococcal disease in infancy.

## Figures and Tables

**Table 1:** Summary of infant’s clinical course.

Hospital Day	Clinical/ Laboratory Findings	Microbiology	Treatment	CSF Findings
0	Fever (38.6 °C), WBC 10.68 K/μL, CRP 7.31 mg/dL.	Blood and urine cultures were obtained	Ceftriaxone at 100 mg/kg/day	
3	WBC 12.65 K/μL, CRP 20.53 mg/dL.	Urine culture: 48,000 CFU/mL of *N. meningitidis* serogroup B. Blood culture of admission flagged positive for *N. meningitidis*	Ceftriaxone continued. Meropenem added	920 WBC/mm^3^, 30,000 RBC/mm^3^
5	Febrile episodes		Ceftriaxone, Meropenem	410 WBC/mm^3^, 3560 RBC/mm^3^
6	Last febrile episode		Meropenem stopped	
11	Discharged in good clinical condition, CRP 0.18 mg/dL		Completing 10 days of intravenous ceftriaxone	
Follow-up at 3 months after discharge	Normal growth and development.			

## Data Availability

The data supporting the findings of this case report are included within the article. No new publicly archived datasets were generated. Additional patient-level data are not publicly available due to patient privacy and confidentiality restrictions but may be made available by the corresponding author upon reasonable request.

## Abbreviations

The following abbreviations are used in this manuscript:

CH50	50% Hemolytic Complement
IMD	Invasive meningococcal disease
FWS	Fever without source
MALDI-TOF MS	Matrix-assisted laser desorption/ionization time-of-flight mass spectrometry
CSF	Cerebrospinal fluid
WBC	White blood cells
RBC	Red blood cells
MRI	Magnetic resonance imaging
CRP	C-reactive protein
CFU	Colony-forming units
